# L’arthroplastie totale de la hanche dans le traitement des luxations congénitales de la hanche chez l’adulte: à propos de 15 cas

**DOI:** 10.11604/pamj.2016.25.201.10534

**Published:** 2016-11-29

**Authors:** Abdelghani El Ayoubi, Mohamed Nasri, Ali Krite, Mohamed El Idrissi, Mohamed Shimi, Abdelhalim El Ibrahimi, Abdelmajid Elmrini

**Affiliations:** 1 Service de Chirurgie Osteo-Articulaire B4, CHU Hassan II, Fès, Maroc

**Keywords:** Arthroplastie, LCH, planification, hypolasie, CROWE, complication, Arthroplasty, LCH, planning, hypoplasia, CROWE, complication

## Abstract

L’arthroplastie totale de la hanche sur luxation congénitale représente un défit pour le chirurgien orthopédiste. Il est maintenant bien établi que le traitement de la maladie congénitale de la hanche chez l’adulte présente un vrai « miracle fonctionnel ». L’évolution des techniques chirurgicales et des matériaux a permis d’élargir les indications de remplacement prothétique jusqu’aux cas les plus complexes allant ainsi à l’encontre de Charnley et Feagin qui écrivaient, en 1973, qu’il n’existait pas de place pour l’arthroplastie totale de la hanche dans les luxations invétérées. Il s’agissait d’une étude rétrospective d’une série de 15 PTH sur luxation congénital de la hanche chez l’adulte, le recueil des données sociodémographiques, cliniques, paracliniques et thérapeutiques s’est fait à l’aide d’une étude des dossiers médicaux des 15 patients et aussi en répondant à un questionnaire au dernier recul. L’âge moyen de nos patients a été de 28 ans; avec une prédominance féminine sex ratio 2F/1H. Il s’agissait d’une dysplasie sévère stade VI selon la classification de crow chez 4 cas, type III chez 9 cas, et seulement 2 cas type II. Tous les patients ont bénéficiés d’une arthroplastie totale de la hanche cimentée, avec anneau de soutien chez 9 cas, et une butté osseuse chez 2 cas. Au dernier recul les résultats fonctionnel selon le score PMA sont excellents et très bon dans 74% des cas. La prise en charge chirurgicale des luxations congénitales de la hanche à l’âge adulte doit obéir et répondre à un cahier de charges lourdes, il s’agissait souvent d’une population jeune et féminine exigeante sur le plan fonctionnel et esthétique. Plusieurs techniques chirurgicales ont été décrites en essayant de résoudre les problèmes liées à cette pathologie, hypoplasie cotyloïdienne et fémorale, l’inégalité des membres inférieurs, etc. L’arthroplastie totale de la hanche sur les luxations congénitales à l’âge adulte reste un challenge pour le chirurgien orthopédiste. C’est une intervention difficile et nécessite une technicité et une programmation et planification particulière pour guetter les incidents indésirables surtout lorsqu’il s’agit d’une population jeune et féminine.

## Abstract (en)

Total hip arthroplasty for the treatment of congenital dislocation represents a challenge for the orthopedic surgeon. It is now well established that the treatment of congenital hip dislocation in adults is a real “functional miracle”. The evolution of surgical techniques and materials has expanded the indications for prosthetic replacement including the most complex cases and thus going against Charnley and Feagin who wrote in 1973 that there was no place for total hip arthroplasty in inveterate dislocations. We conducted a retrospective study of a series of THP 15 for the treatment of congenital hip dislocation in adults;sociodemographic, clinical, paraclinical and therapeutic data were collected from the medical records of 15 patients and also via a written questionnaire which was completed during their last follow-up visit. The average age of our patients was 28 years; female sex ratio was 2F/1H. Severe dysplasia stage VI according to Crowe’s classification was present in 4 cases, type III in 9 cases and type II only in 2 cases. All patients underwent cemented total hip arthroplasty, a reinforcement ring was used in 9 cases and a bone graft in 2 cases. At last follow-up visit PMA functional scores were excellent and very good in 74% of cases. Surgical treatment of congenital hip dislocations in adults must meet strict health standards as congenital hip dislocations often occurs in young female population that is more demanding as to the aesthetic and functional outcomes. Several surgical techniques have tried to solve the problems related to this disease, acetabular and femoral hypoplasia, leg length inequality. Total hip arthroplasty for the treatment of congenital dislocations in adults remains a challenge for the orthopaedic surgeon. This is a difficult surgical procedure which requires technical skills and careful pre-design programming to reduce the occurrence of adverse events especially in the particular case of young female population.

## Introduction

L’arthroplastie totale de la hanche sur dysplasie revêt un caractère particulier en raison du terrain sur lequel elle est réalisée. Il existe toujours, à des degrés divers, une hypoplasie iliaque et fémorale, un retentissement fonctionnel et anatomique sur le rachis et le genou, un déséquilibre de tension des muscles péri articulaires, et une inégalité de longueur des membres inférieurs. Implanter une prothèse totale de la hanche sur ce terrain doit répondre un cahier de charge lourd en redonnant au patient une hanche indolore et stable et mobile, rétablir la longueur des deux membres inférieurs et l’équilibre musculaire, c’est un véritable challenge pour le chirurgien orthopédiste qui est confronté à deux impératifs: il s’agit d’une intervention difficile et nécessite une technicité et une programmation particulière; d’autre part, il s’agit le plus souvent d’un terrain jeune et de sexe féminin exigent sur le plan fonctionnel et esthétique.

## Méthodes

Durant la période étalée entre janvier 2009 et décembre 2013, 15 patients ont bénéficié d’une arthroplastie totale de la hanche sur une luxation congénitale de la hanche. Tous nos patients ont été revus avec un recul moyen de 36 mois. L’étude des dossiers médicaux des patients a permis le recueil des données sociodémographiques, cliniques, paracliniques et thérapeutiques et évolutives et leur analyse a été effectuée à l’aide du logiciel EPI info version 3.5.1.

## Résultats

**Figure 1:**
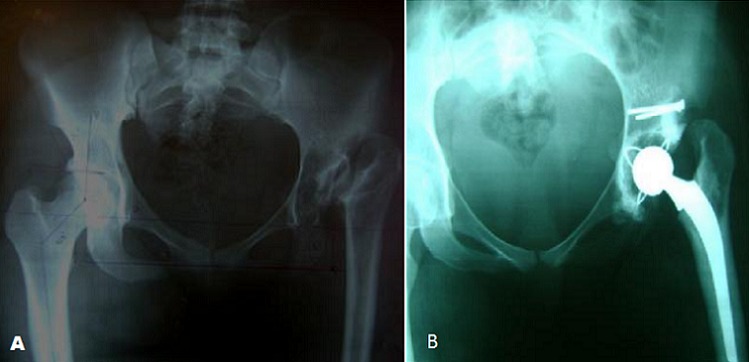
A) Séquelles de LCH gauche chez une patiente de 54 ans; B) Contrôle postopératoire après PTH cimentée et butée du cotyle

**Figure 2:**
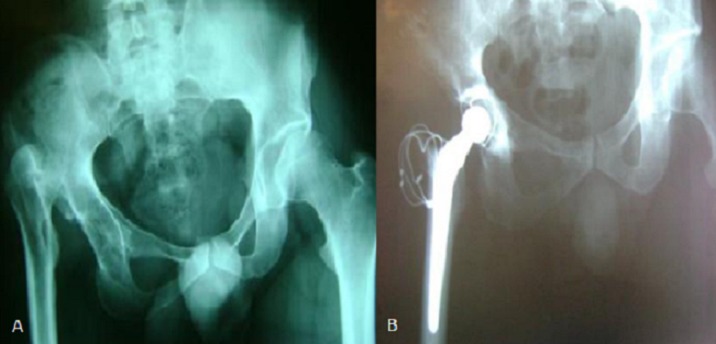
A) LCH droite type III de Crow chez une patiente de 24 ans; B) Contrôle postopératoire après reconstruction acétabulaire avec mise en place d’une cupule cimenté

L’âge moyen de nos patients a été de 28 ans, avec une prédominance féminine sex ratio à 2. La douleur et l’inégalité des membres inférieurs ont été les signes fonctionnels les plus retrouvés, une boiterie type de Trendelenburg a été retrouvée chez plus de 75% des cas, un rachis scoliotique chez deux cas, un raccourcissement de membre inférieur a été présent chez tout nos patients et variable d’un à 5cm. L’évaluation fonctionnelle préopératoire a été effectuée selon le score fonctionnel de Postel et Merle d’Aubigné (PMA) qui a été en moyen de 8. L’analyse radiologique des hanches a trouvé que 4 cas présentaient une dysplasie sévère stade 4 selon la classification de CROWE et 9 cas un stade 3 et seulement deux cas ont présenté un stade 2 (Tableau 1). Tous les patients ont bénéficié d’une arthroplastie de la hanche par la voie d’abord postéro-externe de MOORE. Une trochantérotomie a été effectuée chez 3 cas. Le scellement de l’implant cotyloïdien a été fait par l’intermédiaire d’un anneau de soutien type croix de KERBULL chez 9 cas, un fond de cotyle métallique a été implanté au niveau du paléocotyl chez 3 cas, une ostéotomie fémorale a été réalisée chez 3 cas (Figure 1 et Figure 2). L’évaluation fonctionnelle de nos patients a été effectuée selon le score de PMA au dernier recul qui a été excellent et très bien chez plus de 80% des cas.

**Tableau 1:** Répartition des cas selon le degré de dysplasie selon CROWE et al.

stades	I	II	III	IV
nombre de cas (%)	0 (0)	2(7)	9 (66)	4 (27)

## Discussion

La luxation congénitale de la hanche est définit comme étant un développement anormale de l’articulation de la hanche, caractérisé par des altérations anatomiques impliquant à la fois le cotyle et le fémur. Malgré un traitement bien codifié chez l’enfant et l’adulte jeune [1, 2], le chirurgien orthopédiste se trouve encore confronté au traitement de coxarthrose sur dysplasie de la hanche à l’âge adulte. L’arthroplastie de la hanche représente actuellement un miracle fonctionnel à une population jeune qui souffre de douleur et handicap lié à cette pathologie [1]. Néanmoins, cette procédure est techniquement difficile et associée à des taux élevés de complications, et sa réussite suppose une connaissance parfaite de la pathologie complexe de ces patients, des implants prothétiques adaptés à leur morphologie et une expérience approuvée de l’arthroplastie totale. Un remplacement prothétique de la coxo-fémorale sur LCH est indiqué surtout dans le cadre d’une coxarthrose de la néo-articulation, et dans les formes haute non appuyées, ainsi que les symptômes rachidiens et du genou peuvent participer à cette indication [3–6]. Il faut savoir refuser d’opérer un sujet trop âgé, en médiocre état général et surtout à ceux dont un équilibre psychique trop fragile rend incapables de faire face à une aventure longue et pénible. Il est également fort risqué de céder à la seul préoccupation esthétique- sans la considérer négligeable- elle ne constitue pas toujours une motivation réfléchie, ni une justification suffisante [2].

L’effectif de notre étude est limité, et ne permet pas d’effectuer des analyses statistiques, mais cela ne diminue pas l’intérêt de la question de traitement de séquelles de LCH chez l’adulte. Plusieurs études intéressées à ce sujet [7–12], ont montré que la luxation congénitale de la hanche (LCH) est responsable d’une survenue précoce de coxarthrose. L’âge moyen de nos patients a été de 28 ans, représente une population jeune et active, avec une prédominance féminine [7–11]. Seulement 13% des cas de notre série ont eu un passé chirurgical de la hanche intéressée. Musset [6] a rapporté un pourcentage de 40% sur études de 165 PTH, pour Flecher [7] il a été de 41%, ceci peut être expliqué par la méconnaissance de la pathologie à l’âge de l’enfance dans notre contexte marocain. Les indications de l’arthroplastie dans le traitement de LCH ont été: la douleur sévère, l’impotence fonctionnelle avec difficulté à la marche et à exécuter des activités quotidiennes [4–6], ainsi que les symptômes rachidiens et du genou. Le score de Postel et Merle d’Aubigné représente la cotation la plus adaptée pour l’évaluation fonctionnelle pré et post opératoire de la hanche à opérer. Il est de 3; 3; 2 dans notre série de 15 LCH opérée (Tableau 2). Musset a trouvé, dans une étude faite sur 117 patients, un score moyen préopératoire côté de 3, 3, 2 jugé comme mauvais. Hartofilakidis G et al [13]; dans leur série; ont jugé l’état fonctionnel des hanches opérées comme médiocre. La gravité radiologique a été évalue chez nos patients selon la classification de CROWE et al (Tableau 3).

**Tableau 2:** Évaluation fonctionnelle des résultats selon le score de PMA pré et postopératoire

Score PMA (pts)		<9	10-12	13-14	15-16	17	18
Appréciation		mauvais	médiocre	passable	bon	Très bon	excellent
préopératoire	Nbre de cas	5	6	3	1	0	0
	%	33%	40%	20%	7%	0%	0%
Au recul	Nbre de cas	0	0	2	2	7	4
	%	0%	0%	13%	13%	48%	26%

**Tableau 3:** Évaluation de degré de dysplasie selon la classification CROWE

Auteur	Nombre de cas	Stade I	Stade II	Stade III	Stade IV
Flecher et al	257	39%	30%	14%	17%
Bataga et al	168	0%	19%	52%	29%
Attilla et al	61	11,5%	44,3%	37,7%	6,6%
Pierchon et al	36	39%	19%	22%	20%
Notre série	15	0%	7%	66%	27%

L’arthroplastie totale de la hanche dans le traitement d’une LCH à l’âge adulte sous-entend une stratégie préopératoire et une planification dont l’objectif est de retrouver les conditions biomécanique de la hanche optimales, par le choix du positionnement de la pièce acétabulaire, choix de la tige fémorale, et gestion de l’inégalité des membres inférieurs. Plusieurs études et techniques ont été développé pour répondre à ces objectifs, Woolson et Harris en développé la théorie du “High hip center” après échecs d´une série personnelle de prothèses cimentées avec autogreffes sur séquelle de luxation congénitale [14]. En théorie, le placement pelvien anatomique au niveau du paléocotyle de l’implant acétabulaire diminue le risque de descellement prothétique, il a l’avantage du rétablissement d’un centre de rotation de la hanche permettant ainsi une fonction musculaire d’abduction optimale, et la présence d’un stock osseux disponible pour l'ancrage du composant acétabulaire qui est plus grand que lorsqu'il est placé plus haut. Cependant, l'inconvénient principal, en particulier dans le cas de dislocations élevées, est de la nature plus exigeante de la technique et le risque important de lésion du nerf sciatique et le scellement des implants cotyloïdiens [15–19]. Tout positionnement extra anatomique intervient non seulement sur la direction des forces qui s’exercent au sien les implants et l’interface os-matériel, mais également au niveau de l’amplitude même de ces forces. Plusieurs études ont été menées à fin de répondre et résoudre ces problématiques en proposant des différents techniques comme l’utilisation des prothèses cimentées, non cimentées pour le scellement des implants [20- 21], et pour le comblement des pertes de substance osseux lié surtout à l’hypoplasie de l’os iliaque en utilisant une greffe osseuse [10, 11, 14, 15], butté osseuse [22], anneau de soutien [19].

Dans notre série les prothèses ont été cimentées chez 9 cas, la butté a été utilisé comme geste associée chez deux cas, l’anneau de soutien chez 9 cas et les greffes osseuses par des autogreffes chez 5 cas. L’arthroplastie totale de la hanche dans le traitement des séquelles de luxation congénitale de la hanche à l’âge adulte représente une chirurgie lourde et difficile dans les complications sont fréquentes représentées par les descellements cotyloïdien et fémoral, luxations et fractures de fémur. Dans notre série nous avons eu une luxation de prothèse, une fracture du fémur, et un descellement de l’implant cotyloïdien.

## Conclusion

Envisager de pratiquer une arthroplastie de hanche sur luxation congénitale impose d’anticiper dès la consultation de nombreux aspects: analyser le patient et ses attentes et les comparer au résultat réaliste de cette intervention, ainsi que de réaliser une planification préopératoire clinique et radiologique afin d’anticiper la nécessité d’une trochantérotomie, ou l’utilisation de matériel spécifique, d’implants miniaturisés, voire d’une tige sur mesure. Une fois ces étapes passées, le patient peut s’attendre à une intervention efficace, en particulier sur la douleur et la marche, malgré un taux de complication plus important. Malgré l’efficacité et les résultats satisfaisants de la PTH, elle reste un véritable challenge pour le chirurgien orthopédiste qui est confronté à deux impératifs: d’une part l’intervention est difficile et nécessite une technicité et une programmation particulière, d’autre part, il s’agit le plus souvent d’un terrain exigeant.

### Etat des connaissances actuelle sur le sujet

- La coxarthrose sur LCH devient de plus en plus rare vue sa prise en charge initiale à l’âge de l’enfance;
- L’arthroplastie totale de la hanche permet de répondre au besoin de la population souffrante de cette pathologie sous réserve d’une maitrise total de la technique opératoire et surtout la gestion de ses complications;
- Complications fréquentes et leurs prises en charge nécessite une stratégie adapté et bien conduite.

### Contribution de notre étude à la connaissance

- Les techniques chirurgicales utilisées dans cette série rétrospective de 15 prothèses totales sur maladie congénitale de hanche apportent des résultats satisfaisants avec une amélioration significative du score fonctionnel;
- Dans notre expérience, et à la lumière des données de la littérature, la stratégie technique optimale consiste à: utiliser un couple de frottement adapté au terrain; positionner la cupule dans le paléocotyle; utiliser une reconstruction par autogreffe et enfin la décision d’égalisation des membres va dépendre de la souplesse du rachis lombaire.
